# ATG5 as biomarker for early detection of malignant mesothelioma

**DOI:** 10.1186/s13104-023-06330-1

**Published:** 2023-04-24

**Authors:** Marco Tomasetti, Federica Monaco, Olga Strogovets, Luca Volpini, Matteo Valentino, Monica Amati, Jiri Neuzil, Lory Santarelli

**Affiliations:** 1 Department of Clinical and Molecular Sciences, Polytechnic University of Marche, Via Tronto 10A, Ancona, 60126 Italy; 2 Department of Excellence SBSP-Biomedical Sciences and Public Health, Polytechnic University of Marche, Via Tronto 10A, Ancona, 60126 Italy; 3 Mitochondria, Apoptosis and Cancer Research Group, School of Pharmacy and Medical Science, Griffith University, Southport, Qld 4222 Australia; 4 Molecular Therapy Group, Institute of Biotechnology, Czech Academy of Sciences, Prague- West, 252 50 Czech Republic; 5 Faculty of Science and First Faculty of Medicine, Charles University, Prague 2, 128 00 Czech Republic

**Keywords:** Asbestos exposure, Malignant pleural mesothelioma, ATG5, HMGB1, miRNAs, Biomarkers

## Abstract

**Objectives:**

Malignant pleural mesothelioma (MPM) is an aggressive disease with grim prognosis due to lack of effective treatment options. Disease prediction in association with early diagnosis may both contribute to improved MPM survival. Inflammation and autophagy are two processes associated with asbestos-induced transformation. We evaluated the level of two autophagic factors ATG5 and HMGB1, microRNAs (miRNAs) such as miR-126 and miR-222, and the specific biomarker of MPM, soluble mesothelin related proteins (Mesothelin) in asbestos-exposed individuals, MPM patients, and healthy subjects. The performance of these markers in detecting MPM was investigated in pre-diagnostic samples of asbestos-subjects who developed MPM during the follow-up and compared for the three groups.

**Results:**

The ATG5 best distinguished the asbestos-exposed subjects with and without MPM, while miR-126 and Mesothelin were found as a significant prognostic biomarker for MPM. ATG5 has been identified as an asbestos-related biomarker that can help to detect MPM with high sensitivity and specificity in pre-diagnostic samples for up to two years before diagnosis. To utilize this approach practically, higher number of cases has to be tested in order to give the combination of the two markers sufficient statistical power. Performance of the biomarkers should be confirmed by testing their combination in an independent cohort with pre-diagnostic samples.

**Supplementary Information:**

The online version contains supplementary material available at 10.1186/s13104-023-06330-1.

## Introduction

Occupational and environmental exposure to asbestos may have been significantly underestimate [1]. Many individuals have been exposed or are currently exposed to asbestos or to other carcinogenic mineral fibers by means of a combination of occupational and non-occupational challenges, thus resulting in increased risk of asbestos-related diseases (ARDs) such as malignant pleural mesothelioma (MPM). A decline of MPM incidence has been reported in some countries [2]; however, worldwide incidence of ARDs is expected to peak in the coming decades [3]. Predicting the incidence of MPM is difficult due to the considerable variation in the latency time, varying between 10 years and over 50 years between asbestos exposure to diagnosis. At the time of diagnosis, MPM is often detected at its advanced stages. Linked to the late diagnosis, patient survival rates are low (about 8–14 months from the time of diagnosis). Another highly complicating factor is that MPM is resistant to available therapies. Recently, the combination of nivolumab and ipilimumab showed a benefit for mesothelioma patient [4]. Additionally, the use of nivolumab and ipilimumab has been approved as first-line treatment. A strategy that may help patient survival is early diagnosis based on reliable biomarkers.

The mechanism of asbestos-induced carcinogenesis has been extensively studied [5, 6]. Key regulators of asbestos-driven mesothelial cell transformation include survival based on autophagic pathways [7]. Recent reports document a link between autophagy and inflammation in asbestos-induced carcinogenesis [8]. High levels of high mobility group box 1 protein (HMGB1) were found in serum of asbestos-exposed individuals compared to unexposed controls [8, 9], thus supporting its role as potential biomarker for patients with asbestos-related diseases [10–12]. The release of HMGB1 upon asbestos exposure promoted autophagy by inducing the expression of the autophagic marker autophagy-related gene 5 (ATG5) [8]. Similarly, the microRNAs, miR-126 and miR-222 have been identified as miRNAs potentially involved in asbestos-related malignancies [13, 14]. Increased expression of miR-126 and miR-222 was found in asbestos-exposed subjects, and both miRNAs are involved in major pathways linked to cancer development [15].

In the present study, we investigated the serum levels of ATG5 and HMGB1, miR-126 and miR-222, and the specific biomarker of MPM, soluble mesothelin related proteins (Mesothelin), in asbestos-exposed subjects, MPM patients, and control healthy subjects. The performance of these biomarkers in early detection of MPM was evaluated in pre-diagnosis serum samples of asbestos-exposed subjects who developed MPM during the follow-up.

## Methods

### Study population

Between November 2005 and January 2019, a cohort of asbestos-exposed subjects (n = 641) was recruited and periodically monitored at the Department of Occupational Medicine, Polytechnic University of Marche, Ancona, Italy. The asbestos-exposed subjects regularly underwent lung function analysis, chest radiography and high-resolution computed tomography. During the following 15-years, seven asbestos-exposed subjects developed MPM (Figure S1). The pre-diagnostic samples of MPM-derived asbestos-exposed subjects (n = 7, age at blood collection 75.9 ± 4.7 years, interval time between blood collection and time of diagnosis of 22.4 ± 2.6 months) and a sub-population of 33 asbestos-exposed subjects was selected for biomarker analysis. Of the 33 asbestos-exposed subjects, 13 individuals had both ARDs (asbestosis, pleural plaques, pleural thickenings) and other pulmonary diseases (PDs), as such as chronic obstructive pulmonary disease (COPD) and emphysema, while 4 subjects showed only PDs.

Patients with MPM (n = 32) were accepted between 2008 and 2019 at the Clinic of Pneumology and Thoracic Surgery of the Hospital of Ancona, Italy. Tumor staging was performed according to the Sixth Edition of American Joint Commission on Cancer tumor-node-metastasis (TNM) staging system. The medical charts of all patients were reviewed, and the following information was included: age at diagnosis, sex, occupational history, stage of disease, overall survival (OS) and the follow-up period. The control group consisted of healthy subjects (n = 16) recruited from November 2015 to January 2016 by the Department of Occupational Medicine, Polytechnic University of Marche, Ancona, Italy. The subjects were undergoing occupational surveillance and none of them had ever been exposed to asbestos as documented by their occupational histories. According to Ferrante and colleagues [16], a “fiber-year” exposure metric was calculated for each asbestos-exposed individual, assigning to each person an arbitrary coefficient of “inhaled fibers (ff)” indicating the occupational hazard. Blood was collected at the time of enrolment and periodically during the follow-up period, and serum prepared and stored at -80 °C until use.

### HMGB1, ATG5 and Mesothelin assay

The levels of HMGB1, ATG5 and Mesothelin were assessed using a sandwich-type ELISA assay (My BioSource, MBS771887 for HMGB1 and MBS7209535 for ATG5; Mesomark for Mesothelin) according to the manufacturer’s instructions, and the results were expressed in ng/ml or nM.

### Circulating miRNA assay

The miRNAs were isolated from serum samples as previously described [17], and reverse transcribed to cDNA using the TaqMan Advanced miRNA cDNA Synthesis Kit (Applied Biosystems; Life Technologies) according to the manufacturer’s instructions. The qRT-PCR reactions were carried out using TaqMan Fast Advanced Master Mix (Applied Biosystems; Life Technologies) by using Realplex Mastercycler epgradient S (Eppendorf). The exogenous control (Cel miR-39) was used for normalization and the results were expressed as 2^−ΔCt^.

### Statistical analysis

Results are expressed as mean ± SD unless indicated otherwise. Comparisons between and among groups of data were made using two-tailed Student t-test and one-way ANOVA with Tukey post hoc analysis, respectively. Correlations were performed according to the Pearson test. The receiver operating characteristics (ROC) curves were plotted to quantify the biomarker performance to distinguish asbestos-exposed or non-exposed healthy subjects from subjects with pre-malignancy features. The area under curve (AUC) indicates the average sensitivity of a biomarker over the entire ROC curve, and the maximum Youden Index has been used for calculation of sensitivity and specificity; Backward stepwise logistic regression model with Wald statistical analysis was used to select biomarkers. The predicted probability of being asbestos- exposed subject and asbestos-exposed subject with MPM were used as surrogate biomarkers to construct ROC curves. Kaplan-Meier survival plots and log-rank tests were used to assess differences in survival of MPM patients according to the biomarker cut-offs. Differences with p < 0.05 were considered statistically significant. All data generated in this study were analysed using the SPSS software.

## Results

The study population consisted mainly of males (92%), with mean age of 69.1 ± 10.6 years, of which 86% were non-smokers or former smokers. Radiographic evidence of asbestosis and/or pleural plaques was found in 67% of asbestos-exposed subjects and in 85.2% of MPM patients. MPM was mostly of the epithelioid phenotype (62%) with the OS of 14.6 ± 9.8 months. The demographic, clinical and pathological characteristics of the study cohort are summarized in Table 1.

**Table 1:** Demographic and clinic-pathological characteristics of the study population

	Ctrl (n = 16)	Exp (n = 33)	MPM (n = 32)	Pre-diagnostic samples (n = 7)	Total (n = 88)	p-Value
**Age** (years)	60.8 ± 17.8	70.2 ± 7.7	70.7 ± 6.7	75.9 ± 4.7	69.1 ± 10.6	**p = 0.002**
**Gender** (M/F)	13/3	32/1	29/3	7/0	81/7	p = 0.228
**Smoking** (n/%) Yes No Former	6 (38) 5 (31) 5 (31)	1 (4) 16 (48) 16 (48)	5 (16) 11 (34) 16 (50)	0 (0) 5 (71) 2 (29)	12 (14) 37 (42) 39 (44)	**p = 0.025**
**Asbestos exposure** Duration work (years) Cf (ff/l) × years ARD (Yes/No/ND) PD (Yes/No/ND)	- - - -	25.9 ± 10.3 3.5 ± 5.8 22/11/0 17/16/0	24.0 ± 12.5 5.8 ± 7.6 11/2/19 1/11/20	19.0 ± 1.79.3 ± 8.4 7/0/0 1/6/0	25.0 ± 11.4 4.5 ± 6.7 40/13/19 19/33/20	p = 0.215 p = 0.057 **p = 0.001** **p = 0.0005**
**Histotypes** (n/%) Epithelial Biphasic Sarcomatoid			19 (62) 6 (19) 6 (19)	4 (57) 0 (0) 3 (43)	23 (60) 6 (16) 9 (24)	
**Overall survival** (months)	-	-	14.6 ± 9.8			

Cf, Cumulative fibers; ARDs, asbestos-related diseases; PDs, pulmonary diseases

**Fig. 1:**
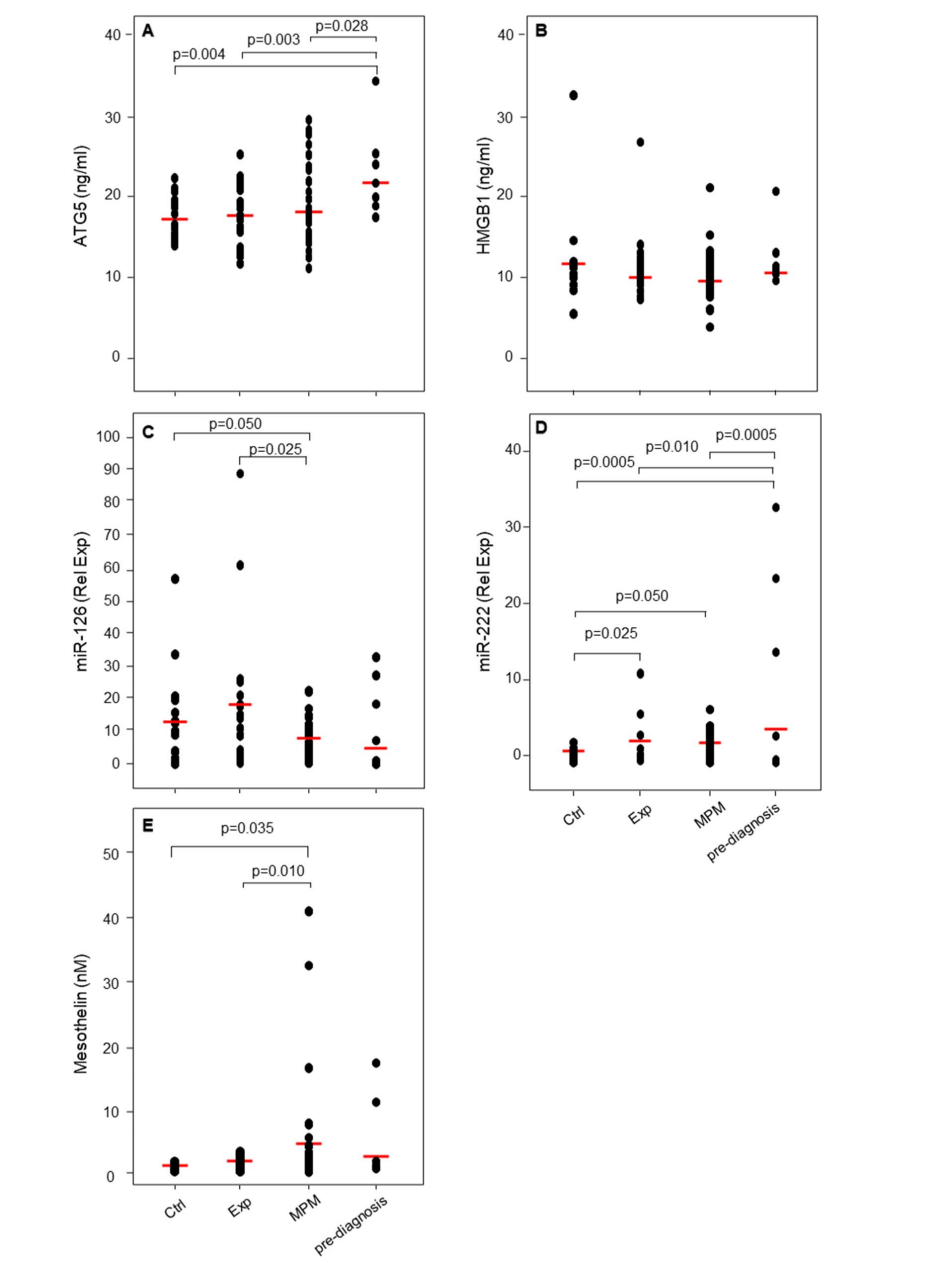
Distribution of biomarkers in the study groups. Serum levels of ATG5, HMGB1, miR-126, miR-222 and Mesothelin in healthy controls (Ctrl), asbestos-exposed subjects (Exp), pre-diagnostic samples, and malignant pleural mesothelioma (MPM) patients. Comparisons among groups were determined by one-way ANOVA with Tukey post hoc analysis. Differences with p < 0.05 are statistically significant

As shown in Fig. 1, the inflammatory and autophagic biomarkers (ATG5 and HMGB1) were not able to discriminate the asbestos exposed and MPM groups from control group. No changes in serum level of ATG5 (Fig. 1A) and HMGB1 (Fig. 1B) was observed among groups. On the other hand, low levels of miR-126 were found in patients with MPM when compared with asbestos-exposed subjects and control group (Fig. 1C), while high levels of miR-222 were detected in serum of asbestos-exposed subjects and MPM patients compared to healthy controls (Fig. 1D), thus suggesting its role as biomarker of exposure. As previously reported, the mesothelin were highly expressed in MPM patients (Fig. 1E).

**Fig. 2:**
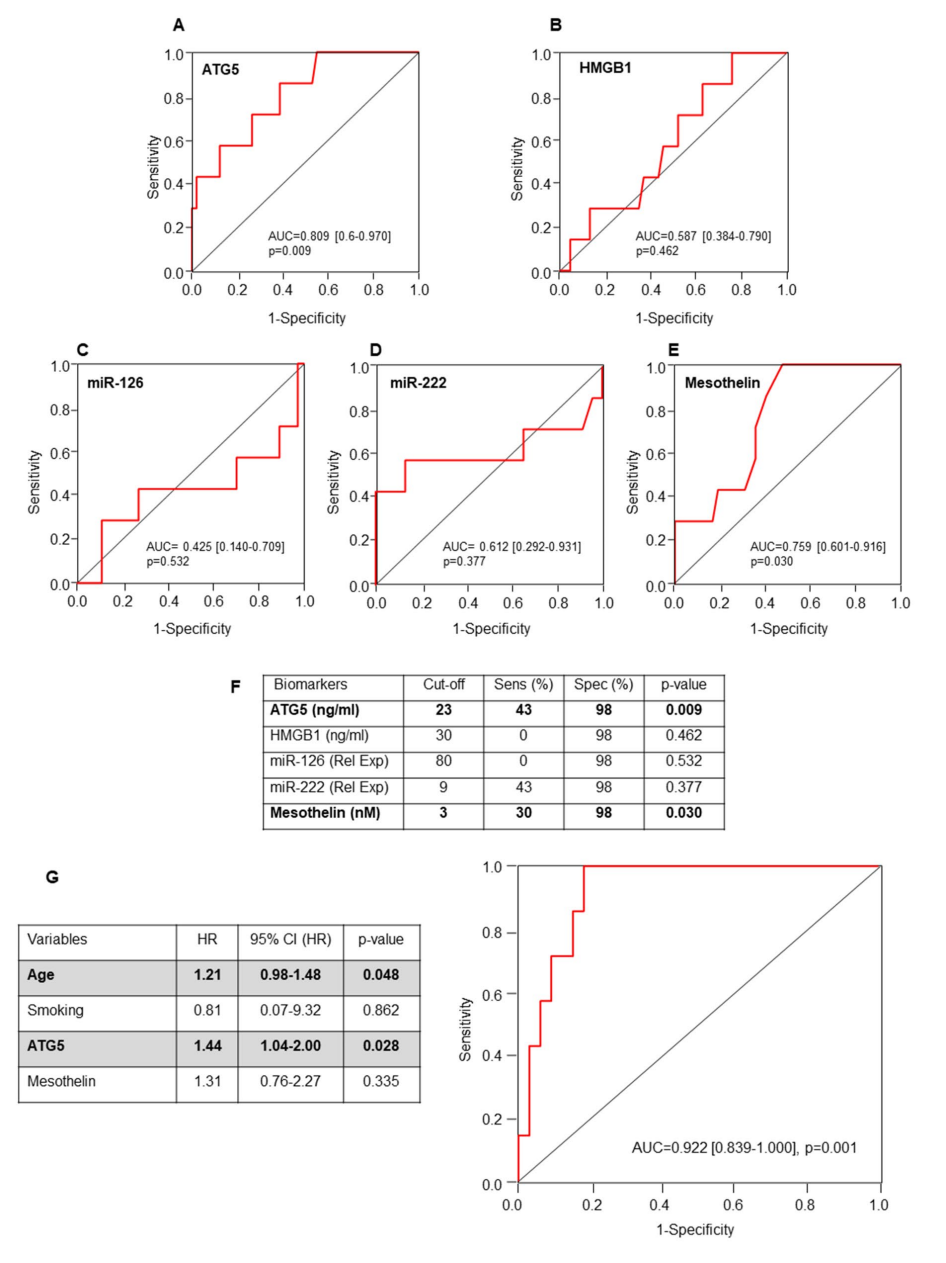
ROC curves and the area under curve (AUC) with confidence interval (CI) of ATG5 (**A**), HMGB1 (**B**), miR-126 (**C**), miR-222 (**D**), Mesothelin (**E**). Cut-offs, sensitivity and specificity are shown (**F**). ROC curve and AUC estimation of the logit model with ATG5 and age using the dataset to discriminate the asbestos-exposed subjects from asbestos-exposed subjects with malignancy (**G**). Differences with p < 0.05 are statistically significant

To investigate the clinical role of these biomarkers in early detection of MPM, their levels were then evaluated in pre-diagnostic samples, which consisted of serum collected prior to the clinical diagnosis (interval time between blood collection and time of diagnosis of median 22 [20–28, minimum-maximum] months). Notably, higher ATG5 and miR-222 levels were found in the pre-diagnosis samples when compared with the asbestos-exposed and control groups (Fig. 1A, D). Although not significant, the pre-diagnosis samples showed elevated levels of mesothelin (Fig. 1E), while no changes for HMGB1 have been observed between the groups (Fig. 1B). Indeed, high levels of ATG5, miR-222 and mesothelin were associated with the presence of benign ARDs, such as asbestosis and/or pleural plaques (Table 2). The ROC analysis of the biomarkers revealed that ATG5 and mesothelin were able to discriminate between pre-diagnosis samples and asbestos-exposed subjects yielding area under the curve (AUC) of 0.809 (95% CI, 0.65–0.97), and 0.759 (95% CI, 0.60–0.92), respectively (Fig. 2A-E**)**. The biomarker cut-offs, sensitivity and specificity were shown in Fig. 2F. The reliability of the analysis was further confirmed by the confusion matrix evaluation (Figure S2). Next, a backward stepwise logistic regression model with Wald statistical analysis was applied to estimate the probability of being asbestos-exposed and developing MPM using data from significant biomarkers (ATG5 and Mesothelin) as cut-off values from ROC curves including age and smoking as confounding variables. A classifier’s optimal logit (P) model was obtained to discriminate the asbestos-exposed subjects from asbestos-exposed subjects with MPM. The predicted probability from the logit model based on ATG5 and age was used to construct the ROC curve (Fig. 2G).

**Table 2:** Biomarker levels according to asbestos-related diseases (ARDs)

	non-ARDs	ARDs	p-value
**ATG5 (ng/ml)**	**17.4 [11.9–23.3]**	**18.6 [12.6–34.2]**	**0.030**
HMGB1 (ng/ml)	11.1 [3.9–32.5]	11.0 [7.3–20.7]	0.470
miR-126 (Rel Exp)	12.8 [0.04–98.2]	9.5 [0.08–60.9]	0.300
**miR-222 (Rel Exp)**	**0.65 [0.01–6.3]**	**2.1 [0.002–33.1]**	**0.033**
**Mesothelin (nM)**	**0.3 [0.1–1.6]**	**1.1 [0.001-32.0]**	**0.050**

**Fig. 3:**
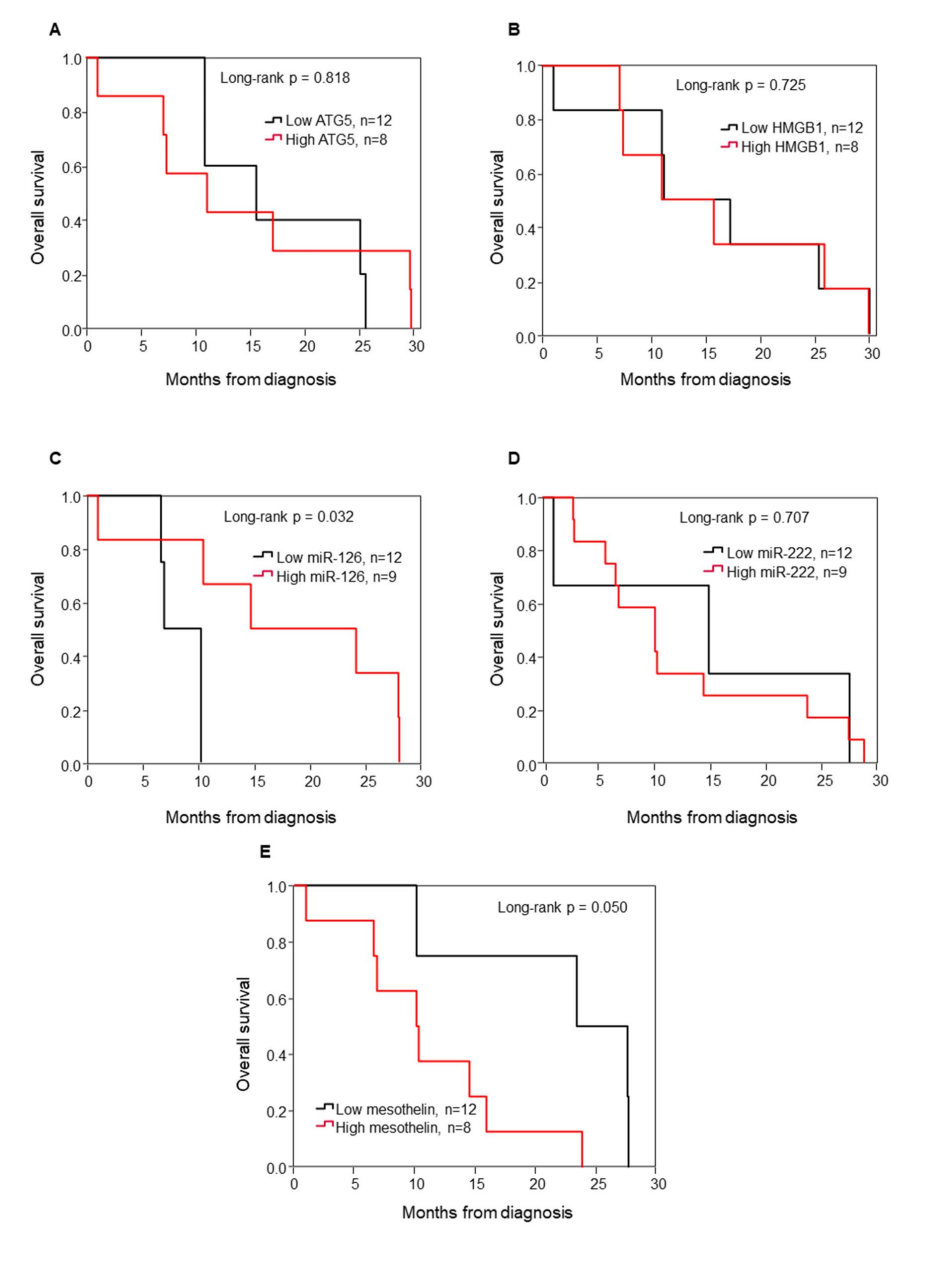
Kaplan-Meier survival curves for e-MPM stratified for the biomarkers. Low and high levels of ATG5 (**A**), HMGB1 (**B**), miR-126 (**C**), miR-222 (**D**) and Mesothelin (**E**) were associated with overall survival (OS). Comparisons between groups were made using log-rank test, and two-sided p < 0.05 was considered statistically significant

The prognosis of MPM is to large extent given by its histotype, such that the epithelioid subtype is associated with significant better OS in comparison to the non-epithelioid histotype [18, 19]. Therefore, we performed Kaplan-Meier survival analyses for epithelioid MPM (e-MPM) according to the ROC cut-off values of the ATG5, HMGB1, miR-126, miR-222 and Mesothelin markers. Of these, miR-126 and SMRP levels were strongly associated with OS. Patients with low miR-126 and high Mesothelin levels had significantly worse OS (Fig. 3).

## Discussion

Despite new therapeutic approaches, such as immunotherapy, as first-line treatment of MPM, its mortality remains high [20–27]. Early diagnosis and better prediction of the malignancy are likely to improve therapeutic responses and patient survival. It has been suggested that biomarkers may be used to predict the development of the disease in subjects at high risk or to predict the response to the treatment. Both HMGB1 and ATG5 have been considered as early biomarkers for monitoring occupational workers who have history of exposure to asbestos. High levels of circulating HMGB1 and ATG5 were found in asbestos-exposed subjects as a result of autophagic activation [10]. ATG5 plays a central role in autophagy, initiating formation of autophagosomes and their fusion with lysosomes [28]. Since ATG5 is a marker of autophagy, its detection in the blood of asbestos-exposed subjects may indicate a process of mesothelial cell transformation. The role of ATG5 as a potential early biomarker was evaluated in pre-diagnosis serum of individuals who developed MPM during the follow-up period. In our study, we found high levels of ATG5 in pre-diagnostic serum of asbestos-exposed subjects who developed MPM (median 22 [20–28, minimum-maximum] months from the diagnosis) compared with asbestos-exposed and control subjects. Similarly, high levels of miR-222 were found in pre-diagnostic samples in comparison to asbestos-exposed, MPM and control groups. Although both ATG5 and miR-222 significantly increased in pre-diagnostic samples in comparison to MPM (p = 0.028 and p = 0.0005, respectively), it might be possible, that other factors except early MPM might have an impact on the ATG5 and miR-222 levels.

Conversely, miR-126, which was found down regulated in cancer, was not able to discriminate the asbestos-exposed subjects from the pre-diagnostic samples. This result confirmed a previous study reporting that three miRNAs, including miR-126 failed to detect MPM in pre-diagnostic serum samples [29]. The ROC analysis indicates that only ATG5 best distinguished the pre-diagnosis group from asbestos-exposed subjects, thus suggesting its potential role as early biomarker.

Predictive performance was found for mesothelin, which is the only validated blood-based biomarker with diagnostic and prognostic value for MPM [30]. On the other hand, definitive role of HMGB1 as an early marker has not been resolved, requiring assessment of larger cohorts of subjects. It has been reported that the hyper-acetylated form of HMGB1 accurately differentiates MPM patients from individuals occupationally exposed to asbestos and from unexposed controls [8]. While the pre-therapeutic levels of HMGB1 are not predictive or relevant to be used as a prognostic marker for non-small cell lung cancer (NSCLC) patients, its role as a biomarker for prediction of response to therapy has been reported [31].

It has been shown that autophagy plays an important role in the regulation of cell death and therapy resistance in MPM tumors [32, 33]. Autophagy can serve as a cytoprotective mechanism following treatments of MPM, thus evaluation of autophagic biomarkers can predict the therapeutic response. Since OS is associated by the histotype of MPM, the prognostic value of the biomarkers was evaluated in patients with the epithelioid MPM subtype, which represents majority of cases. As previously reported, miR-126 and Mesothelin were associated with OS in the univariate analysis [34].

Early detection and therapeutic response are crucial for longer OS of patients with MPM. For the detection of early stages of the cancer, the use of circulating biomarkers in pre-diagnosis samples appears a plausible approach as previously reported [35].

### Limitations

The strength of our study is given by the use of pre-diagnostic samples. However, the relatively low number of evaluated cases limits the statistical power of this approach. The biomarker performance needs to be next confirmed in an independent cohort consisting of higher number of subjects based on serial pre-diagnosis samples.

## Electronic supplementary material

Below is the link to the electronic supplementary material.

Supplementary Material 1

Supplementary Material 2

## Data Availability

The datasets during and/or analysed during the current study available from the corresponding author on reasonable request.

## Abbreviations

ARDs: asbestos-related diseases
ATG5: autophagy-related gene 5
AUC: area under curve
Cf: cumulative fibers
COPD: chronic obstructive pulmonary disease HMGB1: high mobility group box 1 protein
MPM: Malignant pleural mesothelioma
OS: overall survival
ROC: receiver operating characteristics
Mesothelin: soluble mesothelin related proteins
