# Supplementary figures and images for: ATG5 as biomarker for early detection of malignant mesothelioma
Source: BMC Res Notes. 2023 Apr 24;16:61. doi: 10.1186/s13104-023-06330-1 (PMC10127310; doi:10.1186/s13104-023-06330-1)

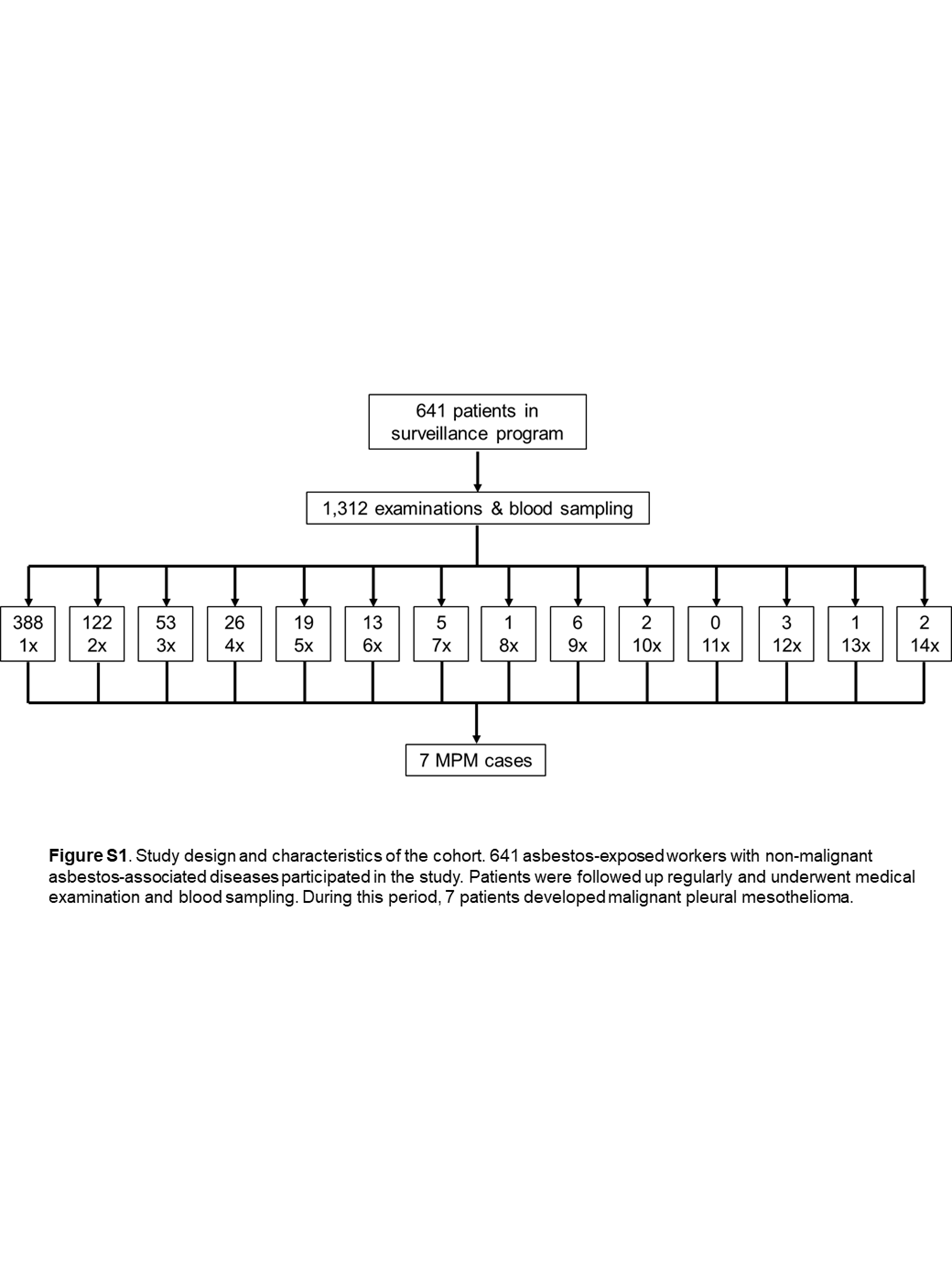

Supplement: Supplementary file 1 — Supplementary Material 1 [file 13104_2023_6330_MOESM1_ESM.tif]

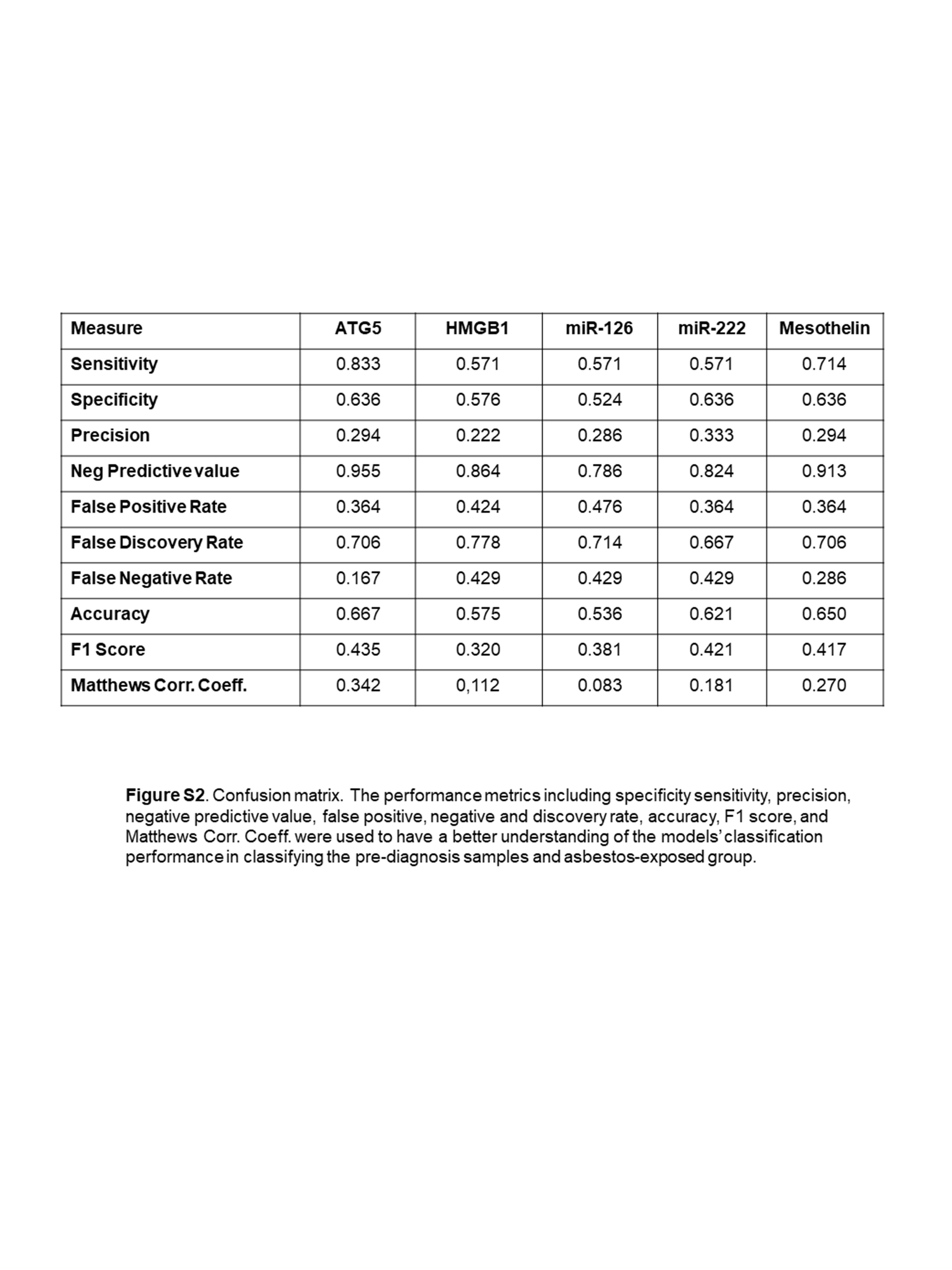

Supplement: Supplementary file 2 — Supplementary Material 2 [file 13104_2023_6330_MOESM2_ESM.tif]
